# An Amidochlorin-Based Colorimetric Fluorescent Probe for Selective Cu^2+^ Detection

**DOI:** 10.3390/molecules21010107

**Published:** 2016-01-18

**Authors:** Wenting Li, Guohua Zhu, Jinghua Li, Zhiqiang Wang, Yingxue Jin

**Affiliations:** Key Laboratory for Photonic and Electronic Bandgap Materials, Ministry of Education, College of Chemistry & Chemical Engineering, Harbin Normal University, Harbin 150025, China; m13039864430@163.com (W.L.); zhuguohua201@163.com (G.Z.); jyxprof@163.com (J.L.)

**Keywords:** fluorescent probe, copper ions, chlorophyll

## Abstract

The design and synthesis of selective and sensitive chemosensors for the quantification of environmentally and biologically important ionic species has attracted widespread attention. Amidochlorin p6 (**ACP**); an effective colorimetric and fluorescent probe for copper ions (Cu^2+^) in aqueous solution derived from methyl pheophorbide-a (MPa) was designed and synthesized. A remarkable color change from pale yellow to blue was easily observed by the naked eye upon addition of Cu^2+^; and a fluorescence quenching was also determined. The research of fluorescent quenching of **ACP**-Cu^2+^ complexation showed the detection limit was 7.5 × 10^−8^ mol/L; which suggested that **ACP** can act as a high sensitive probe for Cu^2+^ and can be used to quantitatively detect low levels of Cu^2+^ in aqueous solution. In aqueous solution the probe exhibits excellent selectivity and sensitivity toward Cu^2+^ ions over other metal ions (M = Zn^2+^; Ni^2+^; Ba^2+^; Ag^+^; Co^2+^; Na^+^; K^+^; Mg^2+^; Cd^2+^; Pb^2+^; Mn^2+^; Fe^3+^; and Ca^2+^). The obvious change from pale yellow to blue upon the addition of Cu^2+^ could make it a suitable “naked eye” indicator for Cu^2+^.

## 1. Introduction

The design and synthesis of selective and sensitive chemosensors for the quantification of environmentally and biologically important ionic species has attracted widespread attention [1]. Among ionic species, copper is one of the important pollution sources [2]. As a common heavy metal existing widely in Nature and all living organisms, an appropriate amount of copper ion is essential to living organisms because it is a key constituent of the respiratory enzyme complex cytochrome c oxidase [3]. However, excess copper ion may cause physical discomfort and sometimes life-threatening illness [4,5,6,7]. Therefore the determination of heavy metal content in living organisms and the environment is particularly important. 

Because chromo- or fluoroionophores are highly effective for these determinations, given their easy handling and the simple equipment required, effort has been expended to develop optical chemosensors that selectively respond to the Cu^2+^ ion. Various methods have been developed in the past decades to determine Cu^2+^ ion content, such as spectrophotometric [8,9,10], electrochemical (EM) [11,12,13], inductively coupled plasma atomic emission spectrometric (ICP-AES) [14,15,16,17], atomic absorption spectroscopic (AAS) [18,19] and fluorescence methods [20,21,22]. Among them, the fluorescence method utilizes a specific chemical reaction between dosimeter molecules and the target species to form a fluorescent or colored product. Thus, high selectivity toward the probe is an advantage of chemodosimeters, making them useful for detecting Cu^2+^ ions. Meanwhile, as paramagnetic Cu(II) ion has a strong ability to quench fluorescence, recent years have seen a growing interest in the development of fluorescent probes for Cu^2+^ with different chemical transducers, such as rhodamine and semiconductor quantum dot-based probes [23,24,25,26,27]. Although rhodamine dyes are widely used as fluorescent probes owing to their high photostabilities, high extinction coefficients, and high fluorescent quantum yields, their structural instability in strong acid or base media (pH < 4 or pH > 9) has limited their applications [28]. Semiconductor quantum dots (QDs) have also emerged as an important class of inorganic nanomaterial that affords promising potential in the ion-detection field, yet QDs probes cannot be applied under alkaline conditions, while the morphology, size and surface defects of the nanocrystals could influence the detection sensitivity [29].

**Scheme 1:**
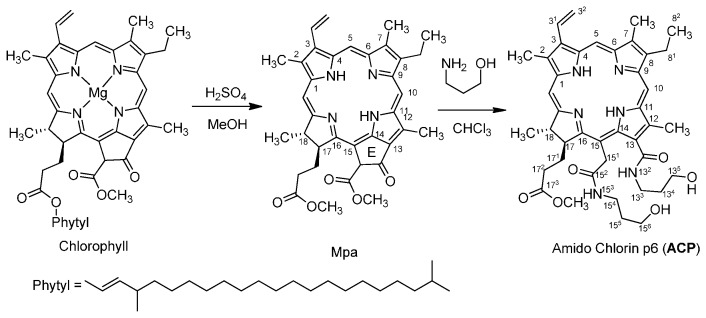
The proposed synthesis of amidochlorin *p*6.

Therefore, the search for new fluorescence probes with sufficient high sensitivity and a wide application range is still an active field as well as a challenge for the analytical chemistry community. Recently, porphyrins have gained widely attention for their good photophysical properties with large Stokes shifts and relatively long excitation (>400 nm) and emission (>600 nm) wavelengths that minimize the effects of the background fluorescence [30]. A newly reported pyro-pheophorbide-a methyl ester (PPME) could selectively complex with Cu^2+^ ions, leading to a distinct change in its absorption spectrum as well as efficient fluorescence quenching [31]. However, the association rate between PPME and Cu^2+^ is very slow, and no systematic research on quantitative detection of Cu^2+^ has been performed. Moreover, the poor water-solubility of PPME had limited its application in sensing Cu^2+^ in aqueous solution, hence improving the water-solubility is necessary and desirable. In this paper, a new chlorophyll-based Cu^2+^ fluorescent probe, amidochlorin p6 (**ACP**), was designed and synthesized. (Scheme 1) Two flexible side chains with hydrophilic hydroxyl groups were introduced to improve the water-solubility of the designed molecule, while the hydroxyl groups may also serve as ligand binding sites to chelate heavy metals. **ACP** has large absorption, strong fluorescence and a relatively long emission wavelength in visible region, displaying high selectivity for Cu^2+^ in aqueous solution among the metal ions examined, with a low detection limit in a wide pH range of 1 to 12. Moreover, **ACP** exhibited marked fluorescence quenching upon the binding of Cu^2+^ ion, thus it has potential applied value for rapid detection of Cu^2+^ in aqueous solution. 

## 2. Results and Discussion

### 2.1. Chemistry

Methyl pyropheophorbide a (Mpa) was synthesized according to the literature procedure [32]. Then propanolamine (1 mL) was introduced to Mpa through a aminolysis reaction of the methyl ester to give the title compound **ACP**.

### 2.2. Recognition of Metal Ion

To verify its metal ion sensing abilities, **ACP** was titrated over a wide range of metal ions, such as Cu^2+^, Zn^2+^, Ni^2+^, Ba^2+^, Ag^+^, Co^2+^, Na^+^, K^+^, Mg^2+^, Cd^2+^, Pd^2+^, Mn^2+^, Fe^3+^, and Ca^2+^. Stock 1 mM solutions of metallic ions were prepared by dissolving the appropriate salts in doubly distilled water, respectively, and then diluting to a lower concentration of 10 µM. Meanwhile, a stock 1 mM solution of **ACP** was also prepared in ethanol, and then diluted to a lower concentration of 10 µM. In brief, to a 10 mL volumetric flask, 100 µL of the stock solution (1 mM) of **ACP** was added, followed by addition of 100 µL of different metal ions stock solutions, the mixtures were diluted to lower concentrations by addition of 50% ethanol (*v*/*v*) solution. As a control, the same procedure was performed but in the absence of Cu^2+^.

### 2.3. Spectral Titration of **ACP** with Cu^2+^

**Figure 1:**
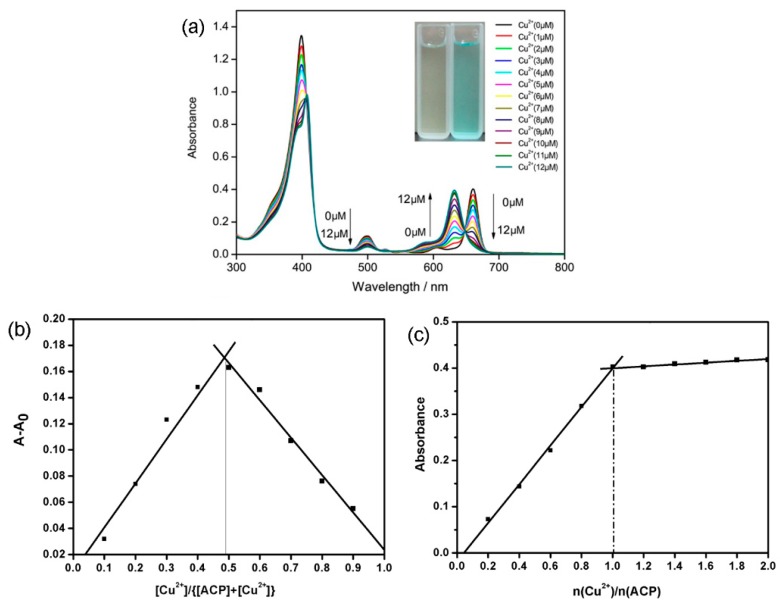
(**a**) The absorption spectrum of **ACP** in water/ethanol (*v*/*v* = 50/50) solution (10 µM) with added Cu^2+^; (**b**) Job’s plot according to the method for continuous variations (the total concentration of **ACP** and Cu^2+^ is 10 μM. The absorbance was measured at 632 nm; (**c**) Mole ratio plot for stoichiometric ratio between **ACP** (10 μM each) and Cu^2+^.

Copper is a quenching metal ion and the coordination of **ACP** with Cu^2+^ would quench the fluorescence of **ACP**. The UV-Vis and fluorescence titration experiments of **ACP** with Cu^2+^ were performed in 50% ethanol (*v*/*v*) solution. Figure 1a shows the UV-visible absorption spectrum of **ACP**. **ACP** absorbs throughout the ultraviolet region into the visible region between about 400 and 800 nm with four peaks: a strong Soret absorption peak at 399 nm, two weak absorption peaks at 499 nm and 605.5 nm, and a Qy peak at 660.5 nm. The absorption of **ACP** is highly affected by the presence of Cu^2+^ ions. Upon addition of Cu^2+^ ions, the absorption intensity of Soret peak at 399 nm decreased with a little red shift, and the peak at 499 nm also decreased, with no peak shift. Meanwhile, the Qy peak gradually reduced in intensity with the formation of a new absorption peak at about 632 nm and with the formation of an isosbestic point at 652 nm. When the concentration of Cu^2+^ increased to the same level as **ACP**, the Qy peak disappeared yet the absorption intensity at 632 nm reached a maximum. The change of **ACP** absorption spectra demonstrated the complexation between **ACP** and Cu^2+^. The value of the shift is indicative of the degree of the interaction between the fluorophore and the bound Cu^2+^. To study the binding stoichiometry of **ACP** and Cu^2+^, a Job’s plot experiment was carried out by using the UV-Vis absorbance spectrum at 632 nm. Keeping the sum of the initial concentration of Cu^2+^ and **ACP** at 10 μM, increasing the concentration of Cu^2+^ from 0 to 1. The maximum absorbance occurred when the [Cu^2+^]/{[**ACP**]+[Cu^2+^]} reached at 0.5.

This observation indicates that **ACP** and Cu^2+^ formed at 1:1 ratio complex. In order to verify this, the mole ratio plot for stoichiometric ratio between **ACP** (10 μM each) and Cu^2+^ was measured. As can be seen from Figure 1c, the molar ratio of **ACP** to Cu^2+^ was 1:1.

**Figure 2:**
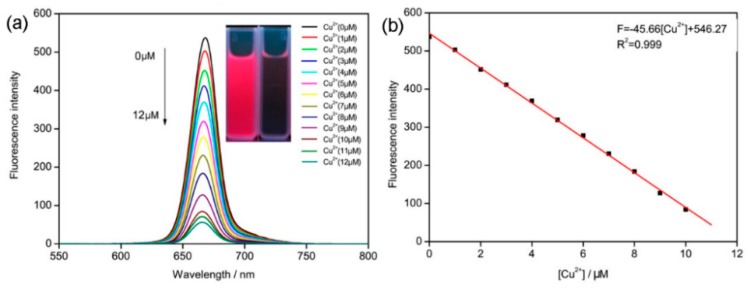
(**a**) Fluorescence spectra of [**ACP**-Cu^2+^] with emission wavelength 668 nm; (**b**) The relation between **ACP** fluorescence intensity and the concentration of Cu^2+^.

The fluorescence titration of Cu^2+^ was carried out using a solution of 10 µM **ACP** in ethanol, using 412 nm as excitation wavelength. As illustrated in Figure 2a, the fluorescence intensity of **ACP** decreases with increasing concentration of Cu^2+^, which constitutes the basis for the determination of Cu^2+^ with the fluorescent probe proposed in this paper. Moreover, it can be seen from Figure 2b that the fluorescence intensity at 632 nm showed a linear quenching with the increasing addition of Cu^2+^. The fluorescent response of **ACP** toward Cu^2+^ was calculated to cover a linear range from 1 to 10 µM. The linear equation was y = −45.66x + 546.27 (R^2^ = 0.999), where y is the fluorescence intensity at 668 nm measured at a given Cu^2+^ concentration and x is the concentration of Cu^2+^ added. The detection limit of Cu^2+^ is 7.5 × 10^−8^ mol/L, which is lower than the limit of Cu^2+^ in drinking water (~20 µM) demanded by U.S. Environmental Protection Agency. This result showed that **ACP** is sensitive enough to monitor the concentration of Cu^2+^ in drinking water.

### 2.4. Selectivity and Interference Studies

**Figure 3:**
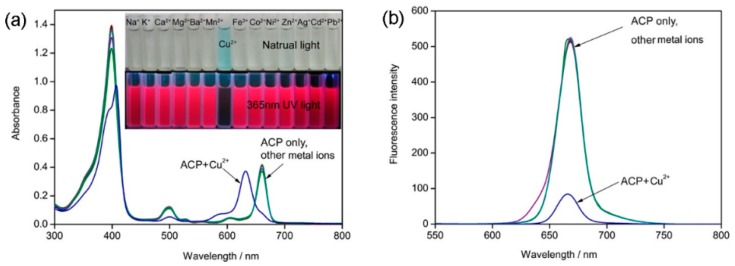
(**a**) UV-Vis absorption and (**b**) fluorescence emission spectra of **ACP** (10 µM) upon addition of various metal ions (10 µM) in water/ ethanol (*v*/*v* = 50/50) solution. The color changes of **ACP** (10 µM) upon addition 1 equiv. of various metal ions under natural light and UV-Vis are also displayed.

**Figure 4:**
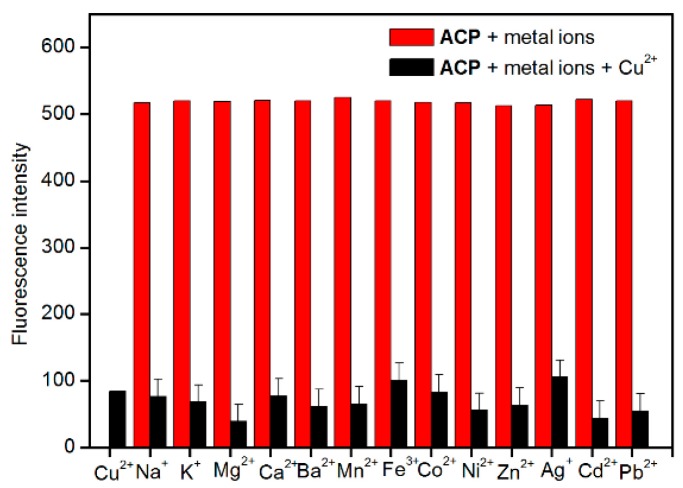
The relative fluorescence intensity diagram of **ACP** (10 μM) to different metal ions (1 equiv.). Excitation was at 412 nm, and emission was at 668 nm.

Selectivity is a very important parameter to evaluate the performance of a probe. Development of chemosensors with “naked eye” capability has an advantage over traditional fluorescence sensors because they do not need cumbersome labor and a sophisticated instruments [33]. The selectivity of **ACP** toward Cu^2+^ and the interference of a number of common ions with the determination of Cu^2+^ were investigated. The experiments were carried out by fixing the concentration of Cu^2+^ at 10 µM and then recording the change of the UV-Vis absorbance and fluorescence intensity before and after adding the interferent into the Cu^2+^ solution (Figure 3). In the presence of other tested metal ions (Zn^2+^, Ni^2+^, Ba^2+^, Ag^+^, Co^2+^, Na^+^, K^+^, Mg^2+^, Cd^2+^, Pd^2+,^ Mn^2+^, Fe^3+^, and Ca^2+^), the UV-Vis absorbance spectra showed almost no obvious change relative to the free ligand **ACP**, and the absorbance of **ACP** was only slightly influenced by the addition of other ions (Figure 3a). When 1 equiv. of Cu^2+^ and selected metal ions (10 µM) was added into the solution of **ACP** (10 µM), many of the investigated metal ions do not interfere with detection of Cu^2+^. The data in Figure 4 clearly reveals that the addition of other common metal ions can hardly affect the fluorescence response of **ACP** towards Cu^2+^. There are only slight interfering effects of Mg^2+^ and Cd^2+^. These results clearly suggest that the probe **ACP** shows a high anti-interference ability against other potentially coexisting metal ions.

Furthermore, upon addition of the same amount of the various metal ions, respectively, only Cu^2+^ induced a striking color change from pale yellow to blue, as observed by the naked eye (Figure 3a). Those observations indicate that **ACP** has a high selectivity to Cu^2+^ and can be a good colorimetric sensor for Cu^2+^ ions. Moreover, upon addition of Cu^2+^ and selected metal ions (10 µM), only Cu^2+^ showed distinct quenching (Figure 3a,b), which suggested that **ACP** can be a selective fluorescent sensor for Cu^2+^ ions. It’s worth mentioning that upon addition of Cu^2+^, the color of **ACP** changed much faster than PPME that previously reported in the literature [31]. In brief, our proposed probe shows extraordinary selectivity to Cu^2+^ and could meet the selectivity requirements for biomedical and environmental applications.

### 2.5. Spike and Recovery Test

Spike and recovery test was conducted in tap water to examine whether there is any positive or negative interference in real drinking water samples. We first examined the effect of tap water on the fluorescence stability and found no quenching effect. The local tap water was filtered first through filter paper to remove any insoluble suspended solids. The recovery study was carried out on a mixture of water and ethanol (1:1, *v*/*v*) which was spiked with 2, 5 and 8 μM Cu^2+^. Each experiment was done in quintuplicate and the average was presented with relative standard deviation. The contents of Cu^2+^ were recovered using the linear equation obtained in Figure 3. The analysis results for the sample with spiked Cu^2+^ were given in Table 1. The result showed that the method had a good recovery at the concentration test, suggesting no serious positive or negative interferences for selectively and sensitively determining copper(II) ion in real water samples.

**Table 1:** Recovery test of Cu^2+^ in tap water ^1^.

Tap Water Sample	Cu^2+^ Added (μM)	Cu^2+^ Found (μM)	RSD (%, *n* = 5)	Recovery (%)
Sample 1	2	2.242	3.75	112.1
Sample 2	5	5.198	1.90	104.0
Sample 3	8	8.404	1.43	105.1

^1^ Values shown were the calculated mean Cu^2+^ for each sample.

### 2.6. Effect of pH

**Figure 5:**
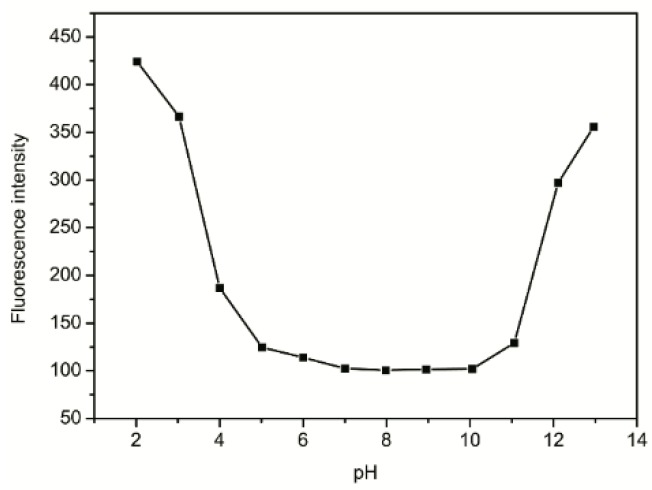
The effect of different pH values on the spectroscopic characteristics of Cu^2+^-**ACP**.

The spectroscopic characters of the probe were studied in the pH range 2–13 in sodium acetate-acetic acid buffer solution. Figure 5 shows the fluorescence response of **ACP** toward Cu^2+^ in the pH range. The fluorescence intensities of the mixture were very high in the pH range 2–4, yet the fluorescence emission (λ_ex_/_em_ = 561/580 nm) drastically decreases in pH up to 5 and varies slightly until 11. This may be attributed to the fact that H^+^ and Cu^2+^ competitively bind to **ACP** in acid solutions, consequently the formation of Cu^2+^-**ACP** complexes are inhibited, thus the mixture displayed high fluorescence intensities in the pH range 2–4. Moreover, in the pH range 11–13 the mixture possess very high fluorescence emission, which is most probably due to the fact that in strongly alkaline solutions OH^−^ and **ACP** competitively bind to Cu^2+^. The more alkaline of the buffer solution is, the more liable it is to form Cu(OH)_4_^2−^, and the more difficult it is to form Cu^2+^-**ACP** complexes. Therefore, the Cu^2+^-**ACP** complexes are stable only in the pH range 6–11.

### 2.7. Binding Mechanism

**Figure 6:**
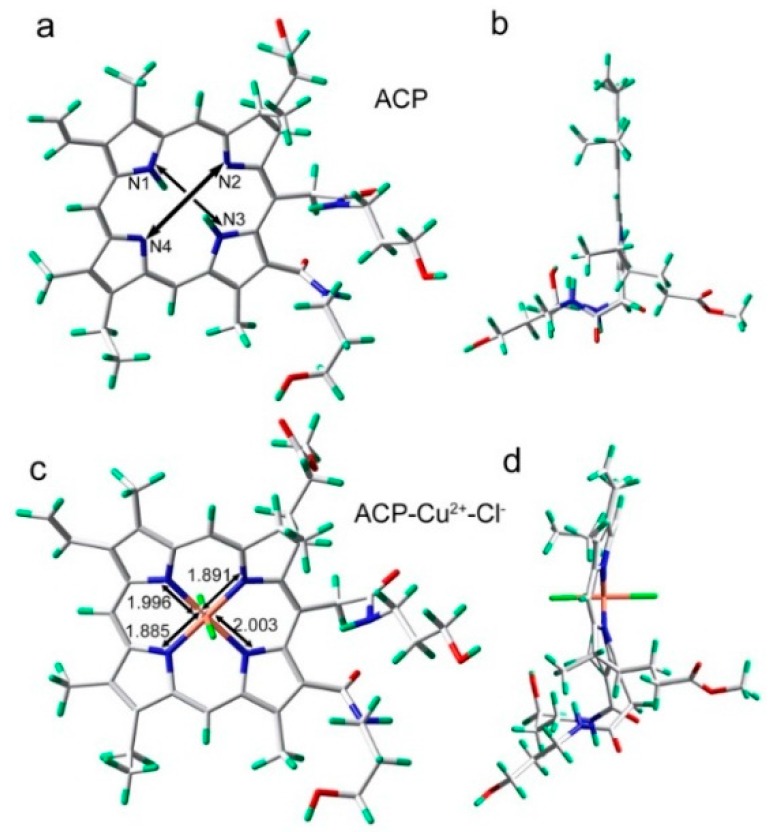
Energy-minimized structures by DFT calculations: (**a**) viewed from the front for ACP; (**b**) viewed from the side for ACP; (**c**) viewed from the front for ACP-Cu^2+^ complex; (**d**) viewed from the side for ACP-Cu^2+^ complex.

As a new kind of porphyrin, **ACP** is endowed with a cyclic π-aromatic system and exhibits unique coordination chemistry. Owing to the four pyrrole units of **ACP**, Cu^2+^ would coordinate with pyrrole N atoms in a square planar shape. We have simulated the **ACP**-Cu^2+^ complex through density functional theory (DFT) calculations with the Becke-3-Lee-Yang-Parr (B3LYP) exchange function using the Gaussian 09 package [34]. The 6-31G (d, p) basis sets were used except for Cu^2+^, where a LANL2DZ effective core potential (ECP) was employed. Figure 6 represents the molecular geometry optimization according to the 1:1 binding stoichiometry of **ACP** with the Cu^2+^ ion. The atom distances of N1-N3 and N2-N4 in **ACP** were 4.272 and 4.115 Å (Figure 6a), respectively, yet in **ACP**-Cu^2+^ complex their distances decreased to 3.999 and 3.776 Å, respectively, which can be attributed to the fact that the electron-donating N atom of pyrrole rings have high affinity to bind to Cu^2+^ with short bond lengths (shown in Figure 6c). Moreover, the four pyrrole rings in **ACP** are almost planar, yet during the formation of **ACP**-Cu^2+^ complex, the Cu^2+^ ions occupy the coordination center of **ACP** and the molecular plane was slightly contorted and metamorphosed due to the formation of coordination bonds and steric strain (Figure 6d). According to the experimental results, the **ACP**-Cu^2+^ complex exhibits an absorption at 632 nm compared with the absorption at 660.5 for **ACP**. This can be easily explained by the above mentioned phenomena: the introduction of Cu^2+^ distorts the conjugate plane of **ACP** molecule, and to some extent destroys the coniugated π-bond of the four pyrrole rings, thus leading to a blue-shift in the UV-visible absorption spectrum of **ACP**-Cu^2+^.

**Figure 7:**
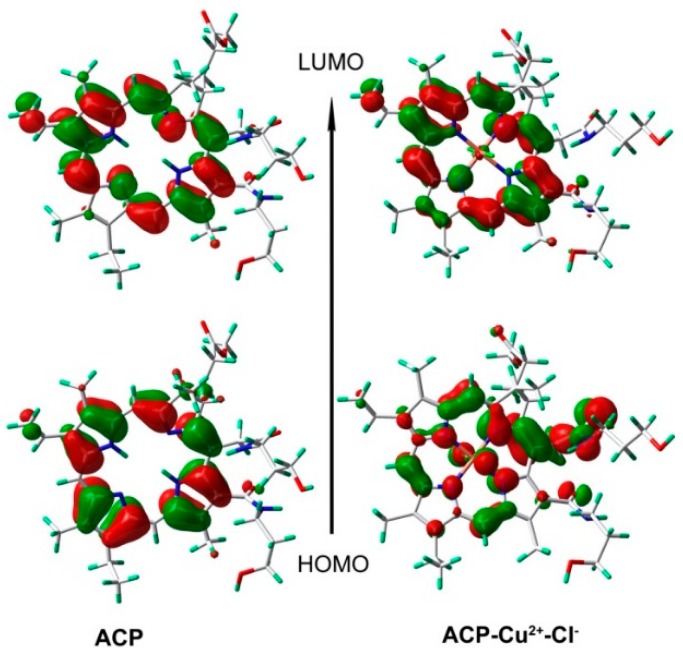
Frontier molecular orbitals of **ACP** and **ACP**-Cu^2+^ complex (1:1) obtained at B3LYP/6-31G(d, p) and B3LYP/LANL2DZ level, respectively.

In addition, we analysed the frontier molecular orbitals (FMO’s) of bare **ACP** and the **ACP**-Cu^2+^ complex. This will help us to understand the quenching phenomenon upon addition of Cu^2+^ ions. The calculated FMO’s are shown in Figure 7. 

In the absence of Cu^2+^, the solution of ACP is pale yellow and fluorescent, and from the figure it is seen that the electron density in HOMO and LUMO are both localized on the pyrrole rings, mainly involving the π-π* electronic transitions of conjugated π-bonds. Upon the addition of Cu^2+^ into the ACP solution, the HOMO of ACP-Cu^2+^ is distributed over the pyrrole rings, Cu^2+^ and amide in the side chain, while in the LUMO the electron density is mainly localized on the pyrrole rings. This is mainly involved in the charge-transfer (CT) from Cu^2+^ and amide in the side chain to the pyrrole rings. Therefore, the quenching phenomenon may be explained by two factors: one is that the coordination of Cu^2+^ to ACP decreases the electron-donating ability of the nitrogen atoms of ACP, and to some extent the conjugated-π bond, whereby the most important ultraviolet absorption and fluorescence were destroyed, resulting in a colour change and fluorescence quenching; the other because of the paramagnetic nature of Cu^2+^. These results support our expectation that ACP could serve as a sensitive fluorescent probe as well as a naked-eye probe for Cu^2+^.

## 3. Experimental Section

### 3.1. General Information

All chemicals used in this paper were obtained from commercial suppliers and used without further purification. Ultrapure water was used for aqueous solution preparation. All samples were prepared at room temperature and promptly used for UV-Vis and fluorescence determination. Zinc chloride (98%), copper(II) chloride dihydrate (99%), nickel(II) chloride hexahydrate (98%), barium chloride (99.5%), silver nitrate (99.8%), cobalt(II) chloride hexahydrate (90%), sodium chloride (99.5%), potassium chloride (99.5%), magnesium chloride hexahydrate (98%), cadmium chloride (99%), lead(II) nitrate (99%), ferric chloride hexahydrate (99%) and calcium chloride anhydrous (96%) were obtained from Sinopharm Chemical Reagent Co. Ltd. (Shanghai, China). All the chemical reactions were performed under argon protection and away from sunshine. ^1^H-NMR and ^13^C-NMR spectra were recorded at 400 and 100 MHz, respectively, on an AMX400 spectrometer (Bruker, Bremen, Germany) with tetramethylsilane (TMS) as an internal standard. Mass spectra were recorded with a VG-7070 spectrometer (Hitachi, Manchester, UK). UV-Vis absorption and emission spectra were recorded using a UV-160A spectrophotometer (Shimadzu, Kyoto, Japan) and spectrofluorophotometer with a 150 W xenon lamp as a visible excitation light source (RF-5301PC, Shimadzu), respectively. All measurements were made at room temperature (about 25 °C). All spectra were obtained in a quartz cuvette (path length = 1 cm). The excitation and emission slit widths were both 10 nm, and PMT voltage of 700 V. The fluorescence intensities/spectra were measured at λ _ex_/_em_ = 412/668 nm.

### 3.2. General Procedure for Synthesis of the title Compound

Methyl pyropheophorbide a (Mpa) was synthesized according to the literature procedure [32]. Then propanolamine (1 mL) was added to a solution of Mpa (66.73 mg, 0.11 mmol) in chloroform and the reaction stirred under a nitrogen atmosphere for 24 h at rt. The reaction mixture was then concentrated, and the residue was dispersed in dichloromethane (30 mL), and then washed by water (30 mL) for three times. After drying and evaporation of the solvent, the residue was purified by silica gel chromatography with methanol: dichloromethane (1:15) as the eluent to give pure **ACP** (76%).^1^H-NMR (CDCl_3_) δ (ppm): 1.00~1.10 (m, 2H, 13^4^-CH_2_), 1.26~1.28 (m, 2H, 15^5^-CH_2_), 1.67 (t, *J* = 7.6 Hz, 3H, 8^2^-CH_3_), 1.68 (d, *J* = 7.2 Hz, 3H, 18-CH_3_),1.73~1.81 (m, 2H, 15^4^-CH_2_), 1.86~1.91 (b, 2H, 13^3^-CH_2_), 2.22~2.28 (m, 2H, 172-CH_2_),2.41~2.45 (m, 2H, 17^1^-CH_2_), 3.27 (s, 3H, 7-CH_3_), 3.48 (s, 3H, 2-CH_3_), 3.51 (s, 3H, 12-CH_3_), 3.68 (s, 3H, 17^3^-OCH_3_), 3.72 (q, *J* = 3.76 Hz, 2H, 8^1^-CH_2_), 3.75~3.88 (m, 4H, 13^5^-CH_2_. 15^6^-CH_2_), 4.31 (b, 1H, 17-H), 4.35 (q, *J* = 7.2 Hz, 1H, 18-H), 4.46 (d, *J* = 7.2 Hz, 3H, 18-CH_3_), 5.29 (d, *J* = 18.5 Hz, 1H, 15-H), 5.39 (d, *J* = 18.5 Hz, 1H, 15-H), 6.13 (d, *J* = 2.8 Hz, 1H, 3^2^-H (Z)), 6.33 (d, *J* = 2.8 Hz, 1H, 3^2^-H (E)), 7.32 (bs, 1H, 13^2^-NH), 8.09 (dd, *J*_1_ = 6.0Hz, *J*_2_ = 11.6Hz, 1H 3^1^-H)), 8.81 (s, 1H, 20-H), 9.62 (s, 1H, 10-H), 9.64(s, 1H, 5-H); ^13^C-NMR (MeOD) δ (ppm): 10.0, 11.5, 11.8, 17.8, 19.8, 23.5, 29.5, 31.8, 32.0, 33.2, 33.3, 33.9, 37.4, 38.6, 38.8, 50.4, 52.8, 54.4, 56.0, 60.4, 60.9, 70.5, 94.7, 98.9, 101.8, 103.5, 121.2, 129.5, 130.5, 130.9, 135.1, 135.6, 136.1, 136.8, 139.6, 145.3, 149.8, 154.8, 171.8, 175.1, 175.2. Anal calcd for C_41_H_52_N_6_O_6_: C 67.93, H 7.23, N 11.59; found C 67.78, H 7.46, N 11.28.

## 4. Conclusions

In summary, we have prepared **ACP**, a simple but effective colorimetric and fluorescent probe for Cu^2+^ detection, from methyl pheophorbide-a. It shows excellent sensitivity and selectivity for Cu^2+^ over other common metal ions in aqueous media. More importantly, the color change upon the addition of Cu^2+^ to **ACP** solutions could make it a suitable “naked eye” indicator for Cu^2+^. Meanwhile, our study of the fluorescence quenching of **ACP**-Cu^2+^ complex showed the detection limit was 7.5 × 10^−8^ mol/L, which suggested that **ACP** can act as a highly sensitive probe for Cu^2+^ and can be used to quantitatively detect low levels of Cu^2+^ in aqueous solution.
